# Intra-tumour molecular heterogeneity of clear cell renal cell carcinoma reveals the diversity of the response to targeted therapies using patient-derived xenograft models

**DOI:** 10.18632/oncotarget.17765

**Published:** 2017-05-10

**Authors:** Baoan Hong, Yong Yang, Sheng Guo, Shayiremu Duoerkun, Xiaohu Deng, Dawei Chen, Shijun Yu, Wubin Qian, Qixiang Li, Qing Li, Kan Gong, Ning Zhang

**Affiliations:** ^1^ Department of Urology, Peking University First Hospital, Institute of Urology, Peking University, Beijing, P.R. China; ^2^ Department of Urology, Beijing Cancer Hospital, Beijing Institute for Cancer Research, Beijing, P.R. China; ^3^ Division of Translational Oncology, Crown Bioscience, Taicang, Jiangsu, P.R. China; ^4^ Department of Urology, Central Hospital of HaMi Region, Xinjiang, P.R. China; ^5^ Department of Urology, People's Hospital of Kelamayi, Xinjiang, P.R. China; ^6^ Center for Cellular & Structural Biology, School of Pharmaceutical Sciences, Sun Yat-Sen University, Guangzhou, P.R. China

**Keywords:** clear cell renal cell carcinoma, molecular heterogeneity, targeted therapy, patient-derived xenograft model, precision medicine

## Abstract

Inter- and intra-tumour molecular heterogeneity is increasingly recognized in clear cell renal cell carcinoma (ccRCC). It may partially explain the diversity of responses to targeted therapies and the various clinical outcomes. In this study, a 56-year-old male ccRCC patient with multiple metastases received radical nephrectomy and resection of the metastatic tumour in chest wall. The surgical specimens were implanted into nude mice to establish patient-derived xenograft (PDX) models with KI2367 model derived from the primary tumour and KI2368 model from the metastastic tumour. The two modles were treated with Sorafenib, Sunitinib, Axitinib, combined Sorafenib/Sunitinib, or alternating therapy of Sorafenib and Sunitinib. Significant anti-tumour activity was found in KI2367 treated with Sorafenib/Sunitinib monotherapy, combined Sorafenib/Sunitinib, and alternating therapy of Sorafenib/Sunitinib (P<0.05) but not in that treated with Axitinib monotherapy. In contrast, KI2368 was significantly responsive to Sunitinib monotherapy, combined Sorafenib/Sunitinib therapy and alternating therapy of Sorafenib/Sunitinib but not responsive to Sorafenib and Axitinib monotherapy (P<0.05). RNAseq of the two models demonstrated that the expression levels of 1,725 genes including the drug targeted genes of *PDGFA*, *PDGFB* and *PDGFRA* were >5-fold higher in KI2367 than in KI2368 and the expression levels of 994 genes were > 5-fold higher in KI2368 than in KI2367. These results suggest the presence of intra-tumour molecular heterogeneity in this patient. This heterogeneity may influence the response to targeted therapies. Multiple biopsy, liquid biopsy and genomic analysis of intra- tumour molecular heterogeneity may help guide a more precise and effective plan in selecting targeted therapies for ccRCC patients.

## INTRODUCTION

Over the past decade, the high-throughput deter-mination of biological molecules such as whole genome sequencing, transcriptome sequencing and proteomics has increased our knowledge about the molecular mechanism of tumourigenesis. On the other hand, the overall incidence of cancers is gradually increasing [1]. The modern concept of cancer treatment emphasizes the reduction of cancer mortality and treatment complications. The second generation sequencing technology has revealed multiple genetic changes in tumours, providing the basis for drug development and personalized cancer treatment. The precision medicine for managing cancers is progressive and attractive. The identification of driver mutations that are critical for tumourigenesis remains a challenge. Several malignant tumours have recently achieved personalized cancer treatment in clinical practice, such as anti-HER2 antibody for *HER2*-positive breast cancer, anti-EGFR therapy for *KRAS* wild-type colon cancer, and BRAF inhibitor for *BRAF* mutant melanoma [2–5].

Renal cell carcinoma (RCC) can be divided into several subtypes according to its histology and molecular differentiation [6–8]. In recent decades, the incidence of RCC has been rapidly increasing at 2.5 percent per year. In 2012, there were about 338,000 new cases of kidney cancer worldwide and an estimated 143,000 cases died of kidney cancer, ranking as the 16th most common cause of cancer death [9–11]. The increased incidence of kidney cancer was partly attributed to the wide use of imaging techniques, such as magnetic resonance imaging (MRI), computed tomography (CT) and ultrasound [12]. Surgery is still the preferred treatment to eradicate the tumour, but approximately 30% of patients undergoing nephrectomy for localized RCC develop metastases [9, 10]. Moreover, RCC is insensitive to chemoradiotherapy. The development of targeted drugs has significantly improved the prognosis of metastatic RCC [13–17]. They target the molecules relating to tumour development to inhibit tumour growth, but few patients got complete or long-term response. Genome-wide studies have confirmed significant genetic diversity among RCCs and found several important driver mutations and multiple of passenger changes in RCCs, which partially explain the heterogeneous clinical outcomes of patients with similar histopathological type [18–21]. In addition to inter-tumour heterogeneity, intra-tumour heterogeneity with diverse genetic subclones within a single tumour has been gradually recognized in RCC [22].

Although a large number of molecular changes in hundreds of renal tumours have been obtained by genome-wide analyses, the effects of these changes on the characteristics of the tumour and clinical presentations have not been clearly elucidated [23, 24]. The therapeutic effect and development of targeted therapies for RCC may be hampered by the inter-tumour and intra-tumour heterogeneity. Single tumour-biopsy may not be able to fully reflect the genetic composition of the tumour due to intra-tumour heterogeneity, causing considerable challenges to the development of individualized precise treatment.

Here we investigated the intra-tumour heterogeneity of gene expression and mutation load in primary and metastatic clear cell renal cell carcinoma (ccRCC) and analyzed the diverse responses to targeted therapies using the patient-derived xenograft (PDX) models. Surgical samples of primary and metastatic ccRCC were collected from a patient. Two PDX models were established to test their responses to targeted therapies. Transcriptome sequencing (RNA-seq) of PDXs was performed to detect the genetic diversity between the primary and metastatic ccRCC.

## RESULTS

### Patient prognosis

The patient was discharged 6 days after the operation. His general status was well after the operation for one month. Afterwards, the patient began to take Sorafenib (400 mg oral, twice daily). Partial response was obtained for his pulmonary metastasis, brain metastasis progressed [25]. The patient died after the operation for 6 months.

### Results of molecular target drugs in treating the kidney carcinoma model of KI2367

**Figure 1:**
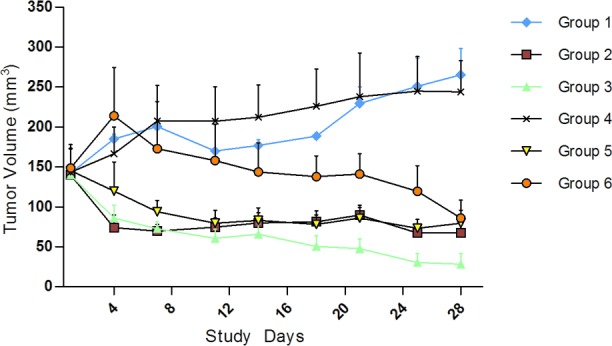
Tumour volume trends for molecular target drugs in treating kidney carcinoma model, KI2367 The result showed obvious anti-tumour activity for Sorafenib/Sunitinib monotherapy, combination and alternating treatment. The tumour volume decreased in all of the above treatment groups following treatment. However, no significant anti-tumour activity with Axitinib monotherapy was seen for the KI2367 model. A significant difference was observed between vehicle/Axitinib and other treatment groups (P<0.05). No significant differences or synergistic tumour growth inhibition was observed between the Sorafenib, Sunitinib, combined therapy and alternating treatment groups. Group 1, Negative Control Group 2, Sorafenib, 50 mg/kg p.o. QD*4 weeks Group 3, Sunitinib, 60 mg/kg p.o. QD*4 weeks, Group 4, Axitinib, 15 mg/kg, p.o. QD*4 weeks, Group 5, Sorafenib 50 mg/kg+sunitinib 60 mg/kg p.o. (QD*4 weeks) Group 6, Sorafenib 50 mg/kg and Sunitinib 60 mg/kg alternated (Weeks 1 and 3, dose with Sunitinib QD, p.o.; Weeks 2 and 4, dose with Sorafenib QD, p.o.).

The KI2367 PDX model was derived from the primary ccRCC tumour. We treated this model with three monotherapies (Sorafenib, Sunitinib, and Axitinib), Sorafenib and Sunitinib combined therapy, and Sorafenib/Sunitinib alternating therapy. The results showed that there was obvious anti-tumour activity from Sorafenib/Sunitinib monotherapy and from combined and alternating treatment of Sorafenib and Sunitinib, with the ΔT/ΔC% values of -66.91%, −46.57%, −238.03% and -60.24% respectively. However, Axitinib showed no significant anti-tumour activity to KI2367, with the ΔT/ΔC% value of 82.11%. A statistically significant difference was observed between vehicle/Axitinib and other treatment groups (P<0.05). No significant difference or synergistic tumour growth inhibition was observed between the 4 effective treatment groups (Figure 1; Table 1).

**Table 1:** The TV and T/C data of applying molecular target drugs in treating kidney carcinoma model KI2367

Treatment	TV^a^ day 0 (mm^3^)^b^	TV post final treatment (mm^3^)	TGI(%)^c^	ΔT/ΔC (%)^d^	P Value^e^
Group 01, Negative control	142.3±5.8	265.3±33.3	—	100	—
Group 02, Sorafenib, 50 mg/kg, Qd*4w, p.o.	140±13.6	67.2±11.5	166.91	−66.91	0.002
Group 03, Sunitinib, 60 mg/kg, Qd*4w, p.o.	144±18.3	28.2±17.1	146.57	−46.57	0.002
Group 04, Axitinib, 15mg/kg, Qd*4w, p.o.	143.3±29.3	244±39.2	17.89	82.11	0.917
Group 05, Sorafenib, 50 mg/kg, Qd*4w, p.o., Sunitinib, 60 mg/kg, Qd*4w, p.o.	145.3±28.2	79.5±16.2	338.03	−238.03	0.001
Group 06, Sunitinib(1,3w), 60 mg/kg, Qd*2w, p.o., Sorafenib(2,4w), 50 mg/kg, Qd*2w, p.o.	148.7±29.5	49±24.9	160.24	−60.24	0.002

a. Total Volume; b. Mean ± SEM; c. Tumor Growth Inhibition (TGI) = (1-%ΔT/ΔC))*100; d. ΔT/ΔC(%) = (mean(T)-mean(T0))/(mean(C)-mean(C0)) * 100%; e. Compare to Vehicle Control.

### Results of molecular target drugs in treating the kidney carcinoma model of KI2368

**Figure 2:**
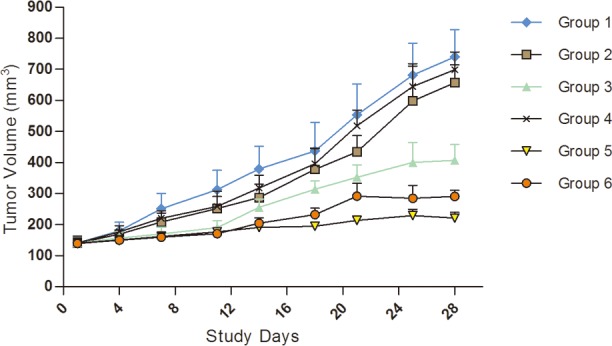
Tumour volume trends for molecular target drugs in treating kidney carcinoma model, KI2368 The result showed obvious anti-tumour activity for Sunitinib monotherapy, Sorafenib and Sunitinib combination or alternating treatment. However, no significant anti-tumour activity for Sorafenib or Axitinib monotherapy was seen in the model. A significant difference was observed between vehicle/Axitinib/Sorafenib and other treatment groups (P<0.05). Synergistic tumour growth inhibition was observed in the Sorafenib and Sunitinib combination therapy groups compared with the drug monotherapy groups (P<0.05). Group 1, Negative Control Group 2, Sorafenib, 50 mg/kg p.o. QD*4 weeks Group 3, Sunitinib, 60 mg/kg p.o. QD*4 weeks, Group 4, Axitinib, 15 mg/kg, p.o. QD*4 weeks, Group 5, Sorafenib 50 mg/kg+sunitinib 60 mg/kg p.o. (QD*4 weeks) Group 6, Sorafenib 50 mg/kg and Sunitinib 60 mg/kg alternated (Weeks 1 and 3, dose with Sunitinib QD, p.o.; Weeks 2 and 4, dose with Sorafenib QD, p.o.).

The KI2368 PDX model was derived from the metastatic ccRCC in left chest wall of the patient. We observed obvious anti-tumour activity from Sunitinib monotherapy and combined and alternating treatments of Sorafenib and Sunitinib, with the ΔT/ΔC% values of 40.09%, 12.25%, and 23.12% respectively. However, there were no significant anti-tumour activities from Sorafenib and Axitinib monotherapy, with the ΔT/ΔC% values of 79.02% and 85.45% respectively. A statistically significant difference was observed between vehicle/Sorafenib/Axitinib and the other treatment groups (P<0.05). Synergistic tumour growth inhibition was observed in the Sorafenib and Sunitinib combined therapy groups compared with the monotherapy groups (P<0.05) (Figure 2; Table 2).

**Table 2:** The TV and T/C data of applying molecular target drugs in treating kidney carcinoma model KI2368

Treatment	TV^a^ day0 (mm^3^)^b^	TV post final treatment (mm^3^)	TGI(%)^c^	ΔT/ΔC (%)^d^	P Value^e^
Group 01, Negative control	140.99±16.78	793.89±87.38	—	100	—
Group 02, Sorafenib, 50 mg/kg, Qd*4w, p.o.	140.23±22.0	656.33±99.5	20.98	79.02	0.654
Group 03, Sunitinib, 60 mg/kg, Qd*4w, p.o.	140.37±17.68	407.00±51.22	59.91	40.09	0.035
Group 04, Axitinib, 15mg/kg, Qd*4w, p.o.	140.9±10.69	698.67±15.68	14.55	85.45	0.912
Group 05, Sorafenib, 50 mg/kg, Qd*4w, p.o., Sunitinib, 60 mg/kg, Qd*4w, p.o.	140.89±11.79	220.67±18.84	87.75	12.25	0.001
Group 06, Sunitinib(1,3w), 60 mg/kg, Qd*2w, p.o., Sorafenib(2,4w), 50 mg/kg, Qd*2w, p.o.	139.43±17.65	290.33±20.17	76.88	23.12	0.001

a. Total Volume; b. Mean ± SEM; c. Tumor Growth Inhibition (TGI) = (1-%ΔT/ΔC))*100; d. ΔT/ΔC(%) = (mean(T)-mean(T0))/(mean(C)-mean(C0)) * 100%; e. Compare to Vehicle Control.

### RNAseq analysis of KI2367 and KI2368 PDXs

The expression of 53598 genes was examined in KI2367 and KI2368 by RNAseq. We found 1,725 genes with > 5-fold higher expression levels in KI2367 than in KI2368; these genes included drug target-related genes such as *PDGFA*, *PDGFB* and *PDGFRA* (Supplementary Table 1). A total of 994 genes had > 5-fold higher expression levels in KI2368 than in KI2367 (Supplementary Table 2). In addition, 5,539 and 5,827 mRNA changes predicted to result in protein variants were found in KI2367 and KI2368 respectively, and 4,023 mRNA changes predicted to result in protein variants were found both in KI2367 and KI2368. The variant detection by RNAseq largely depends on the gene expression level, and it is likely that many of the variants are false positives. We also found 20 and 4 in-frame gene fusions in KI2367 and KI2368, respectively, but no common in-frame fusion was detected (Table 3; see Supplementary Figure 1-5 for validation).

**Table 3:** In-frame gene fusions in KI2367 and KI2368

Sample	Up-gene	Up- chr	Up-Genome- pos	Dw-gene	Dw- chr	Dw- strand	Dw-Genome- pos
KI2367	ACSS1	chr20	24988402	APMAP	chr20	-	24964655
KI2367	ACSS1	chr20	24988404	APMAP	chr20	-	24964654
KI2367	ALDH1A1	chr9	75524545	GAPDH	chr12	+	6647073
KI2367	ASL	chr7	65557650	CRCP	chr7	+	65592691
KI2367	CFI	chr4	110687748	ALDOA	chr16	+	30081492
KI2367	CLCF1	chr11	67140995	POLD4	chr11	-	67120548
KI2367	CP	chr3	148904356	ALDOA	chr16	+	30080934
KI2367	EEF1A1	chr6	74228900	RPL35A	chr3	+	197678056
KI2367	FABP1	chr2	88425695	COL1A2	chr7	+	94045741
KI2367	FGG	chr4	155527982	GAPDH	chr12	+	6646833
KI2367	FTL	chr19	49468781	APOL1	chr22	+	36661638
KI2367	IMMP2L	chr7	111161369	DOCK4	chr7	-	111387499
KI2367	P4HB	chr17	79803483	CALR	chr19	+	13050364
KI2367	P4HB	chr17	79804351	CALR	chr19	+	13054623
KI2367	PKM	chr15	72501143	FTH1	chr11	-	61732905
KI2367	PKM	chr15	72511365	GAPDH	chr12	+	6647094
KI2367	SDHA	chr5	254621	CLPTM1L	chr5	-	1323026
KI2367	SERPINA1	chr14	94844887	CP	chr3	-	148930369
KI2367	SERPINA1	chr14	94844899	EEF1A1	chr6	-	74228848
KI2367	VIM	chr10	17271889	ENO1	chr1	-	8930567
KI2368	C3	chr19	6712322	SPP1	chr4	+	88901222
KI2368	HDAC8	chrX	71571583	CITED1	chrX	-	71522784
KI2368	SDHA	chr5	254621	CLPTM1L	chr5	-	1323026
KI2368	SLC28A1	chr15	85467341	PDE8A	chr15	+	85607601

*PDGFRA* was highly expressed in KI2367, but not expressed in KI2368. The expression level of *PDGFRB* was higher in KI2367 than in KI2368 (Table 4). The frameshift insertion in the *HIF1A* gene was observed in KI2368, which may impact the expression of *PDGFRB* and other genes (Table 5; Supplementary Figure 6). Moreover, a frameshift mutation in *RICTOR* that may affect HIF1A function and subsequently *PDGFR* expression was found in KI2368, and a frameshift mutation in *VEGFB* was detected in KI2368 but not in KI2367 (Table 5; Supplementary Figure 7).

**Table 4:** Gene expression of drug targets and their ligands

	KI2367	KI2368
VEGFA	8.661	10.360
VEGFC	3.053	1.294
VEGFB	6.072	5.821
PDGFB	1.818	−0.118
PDGFRB	−0.675	−4.659
PDGFRA	6.117	−3.478
PDGFC	2.693	3.859
PDGFD	−1.443	−10.483
PDGFA	4.780	−1.381
KDR	−5.978	−6.862
FLT4	−4.530	−5.238
FLT1	−3.897	−5.317
FLT3	−5.274	−8.522
KIT	−0.95918	0.841965

Expression in log_2_(FPKM).

**Table 5:** Gene variants found in MTOR pathway

Gene	KI2367-P9	KI2368-P7
RPTOR	H126Y	not found
PIK3R3	not found	N329K
RICTOR	not found	N1473fs
HIF1A	not found	S190fs
VEGFB	not found	P126fs

*Variants found by RNA-seq.

## DISCUSSION

In recent years, the incidence of RCC has rapidly increased. On the other hand, adjuvant therapies for RCC have also been greatly improved and several randomized trials for RCC have been carried out. The early studies included trials of chemotherapy, hormonotherapy, immunotherapy such as interferon alpha and interleukin-2 [26–32], and autologous vaccination strategies [33–36]. These were the alternatives for treatment at the time but were less effective for metastatic RCC, and the overall outcomes were unsatisfactory [37, 38]. With the development of cancer therapy, we are now in the era of second-generation adjuvant studies [39]. Targeted therapies inhibit the tumour progression by interfering with tumour-associated signaling molecules involved in cancer growth, invasion and metastasis [13, 40–45].

The targeted drugs of tyrosine kinase inhibitors (TKIs) and anti-VEGF antibodies, such as Sorafenib, Sunitinib, Pazopanib and Axitinib are now recommended as the first- or second-line treatment for RCC. Sunitinib has been approved by the United States Food and Drug Administration (FDA) and European Medicines Agency as a first-line treatment for RCC [46]. The mammalian target of kanamycin (mTOR) inhibitor Temsirolimus has been approved for the first-line treatment of RCC patients with poor-prognosis, and Everolimus has been recommended for patients with advanced RCC or unresponsive to anti-VEGF therapies [42]. The targeted therapies have obtained better progression-free survival compared with either placebo, IFN or IL-2 treatment [13, 40–45].

Unfortunately, it is difficult to achieve complete and long-term tumour control with targeted therapy. Tumours may adapt to chronic drug use and escape from drug-mediated growth control [47]. Only a portion of patients responded well to the targeted therapies. With the recognition of more key molecules in the tumourigenic signalling pathway of RCC including VEGF, PDGF and EGF, new agents against these targets have been developed [48]. New targeted drugs such as cabozantinib and lenvatinib have recently been approved for treating advanced renal cell carcinoma [49, 50]. Sequential therapy is considered to be an innovative option that provides maximal efficacy with a minimum risk of therapeutic failure [51, 52]. The agent that is still useful after resistance to a TKI-based regimen remains unclear.

Another hypothesis for the poor long-term effect of targeted therapies is the heterogeneity of RCC. The high-throughput sequencing have identified significant genetic diversity and screened out several important driver mutations and multiple of passenger changes in RCC [53, 54]. The diversity of drug response, resistance to targeted therapies and varied clinical outcomes may be partly attributed to the molecular heterogeneity of RCC.

Inter- and intra-tumour molecular heterogeneity are intriguing and important characteristics of tumours. Intra-tumour heterogeneity with diverse genetic subclones in a single tumour has been gradually recognized in RCC [22]. Nowell reported that subclones have the same origin and share common genetic changes; but each subclone also harbours unique somatic mutations [55]. Gerlinger *et al*. applied next-generation sequencing to characterize intra-tumour heterogeneity in primary and metastatic ccRCC [23, 24]. Genomics analyses from multiple regions of a primary tumour identified a common clonal origin. Somatic mutation and allele imbalance in different subclones showed considerable variability. Of note, the alteration of phosphoinositide 3-kinase (PI3K)–mTOR appeared in different subclones of RCC, possibly relating to resistance to mTOR inhibitors. Gerlinger *et al*. also compared the genetic alterations between primary and metastatic RCCs, and demonstrated that metastatic RCC had genetic features different from those of primary tumour [23, 24]. Therefore, metastatic tumours may originate from rare primary tumour clones, consistent with the findings in other malignancies [56–59].

Our results also indicate that molecular heterogeneity especially the intra-tumour heterogeneity is an important factor for the diversity of response to targeted therapies. This patient had multiple metastases in brain, lung, and skin and underwent right radical nephrectomy. After the operation, the patient *received Sorafenib therapy*. During the follow-up period, metastases in lung became stable but metastases in brain progressed. *Finally, the patient died of brain metastases*. Subsequently, Sorafenib had a diverse effect on metastases at different locations in the same patient. The PDX models of KI2367 and KI2368 showed different responses to Sorafenib monotherapy (in group 2), also indicating that metastases in different locations may have different molecular changes that may relate to the diverse responses to targeted therapies. Consequently, metastatic tumours could have diverse responses and some may become resistant to the targeted therapies[60].

We observed that KI2367 and KI2368 had different response to the TKIs, indicating that primary and metastatic tumours have different genomic profiles. RNAseq analysis reveals that PDGFRA is highly expressed in KI2367 but not expressed in KI2368. The expression of PDGFRB is higher in KI2367 than in KI2368. Conversely, KIT is expressed in KI2368 but not expressed in KI2367. HIF1 is known to induce the transcription of many angiogenesis-related genes. The frameshift insertion in HIF1A may impact on the expression of PDGFRB and other genes in KI2368. The frameshift mutation of RICTOR in KI2368 may also affect the function of HIF1A and subsequently PDGFR expression. It is possible that the difference in PDGFR expression affects the drug responses in KI2367 and KI2368. We also found a VEGFB frameshift mutation in KI2368 but not in KI2367. VEGFB is dispensable for blood vessel growth but is critical for their survival of blood vessels. The VEGFB mutation may reduce the effectiveness of drugs such as Axitinib on VEGF receptors. Sorafenib, Sunitinib and Axitinib target the PDGFR and VEGFR. Axitinib is reported to inhibit KIT and the BCR-ABL fusion protein. Sorafenib also inhibits RAF family kinases, and Sunitinib inhibits KIT, RET, CD114 and CD135. It is likely that the activation of kinases other than PDGFR and VEGFR influences the different efficacy of the three drugs. In KI2368, we observed that the anti-tumour activity of combined or alternating therapies was stronger than monotherapy. The combined or alternating therapies can inhibit more targets and block the tumour growth.

Taken together, these findings confirm again that there is intra-tumour molecular heterogeneity in ccRCC. The intra-tumour heterogeneity could influence the clinical outcome of targeted therapies and explain the diverse drug response among metastatic tumours at different sites from a ccRCC patient. Therefore, intra-tumour heterogeneity may lead to tumour evolution and becomes a huge challenge to the development of personalized therapy for RCC. A single tumour-biopsy may not be able to accurately reflect the features of a tumour. Multiple biopsy, liquid biopsy and genomic analysis could further identity intra-tumour molecular heterogeneity. Multiple biopsies in patients with multiple metastases are not clinically feasible. However, with the development of minimally invasive technology and imaging technology, it will be possible to collect multiple biopsies. Furthermore, liquid biopsy is also a very popular method and may be used in the future. Thereafter, precise and comprehensive results could help us more effectively plan targeted therapies. Further studies are needed to understand the cause of intra-tumour heterogeneity in ccRCC to guide the clinical treatment of patients.

## MATERIALS AND METHODS

### Mice

Female BALB/c nude mice (4-6 weeks, 16-20g, SPF degree) were supplied by Beijing FuKang Bioscience (Beijing, China, Animal Certificate No.: 11401300025891). Procedures related to animal handling, care, and treatment in this study were performed according to the guidelines approved by the Institutional Animal Care and Use Committee (IACUC) of Crown Bioscience following the guidance of the Association for Assessment and Accreditation of Laboratory Animal Care (AAALAC).

### Collection of tumour specimens

The patient was diagnosed with ccRCC at the Department of Urological Oncology of Peking University Cancer Hospital. All protocols in this study were reviewed and approved by the Institutional Medical Ethics Committee, and written informed consent was obtained from the patient. After operation the specimens were examined by pathologists, and some of the samples were used for laboratory study. The specimens for PDX models were preserved in HTK solution (CUSTODIOT, XISAIER Technology, Beijing, China) at 4°C and treated within 6 hours..

### Preparation of PDX of ccRCC

Methods and parameters regarding PDX and tumour inhibition assay using the PDX have been described previously [61, 62]. The specimens were sliced into approximately 3×3×3 mm^3^ fragments (n=20), and 5 mice were implanted with 4 blocks each. The dorsal surface of the mouse was prepped with betadine solution, and the specimens were subcutaneously inoculated into the flanks of mice with a 16-G needle. The tumour growth was monitored twice weekly using a calliper. The established tumour models (passage 0 or P0) were serially re-engrafted to maintain the tumours *in vivo*. These subsequent passages were called P1, P2, P3, etc. When the tumours reached 500–700 mm^3^ (1/2 length x width^2^), they were harvested for the next round of engraftment for serial passage or conducting studies of efficacy and molecular analyses.

### Clinical data of the patient

**Figure 3:**
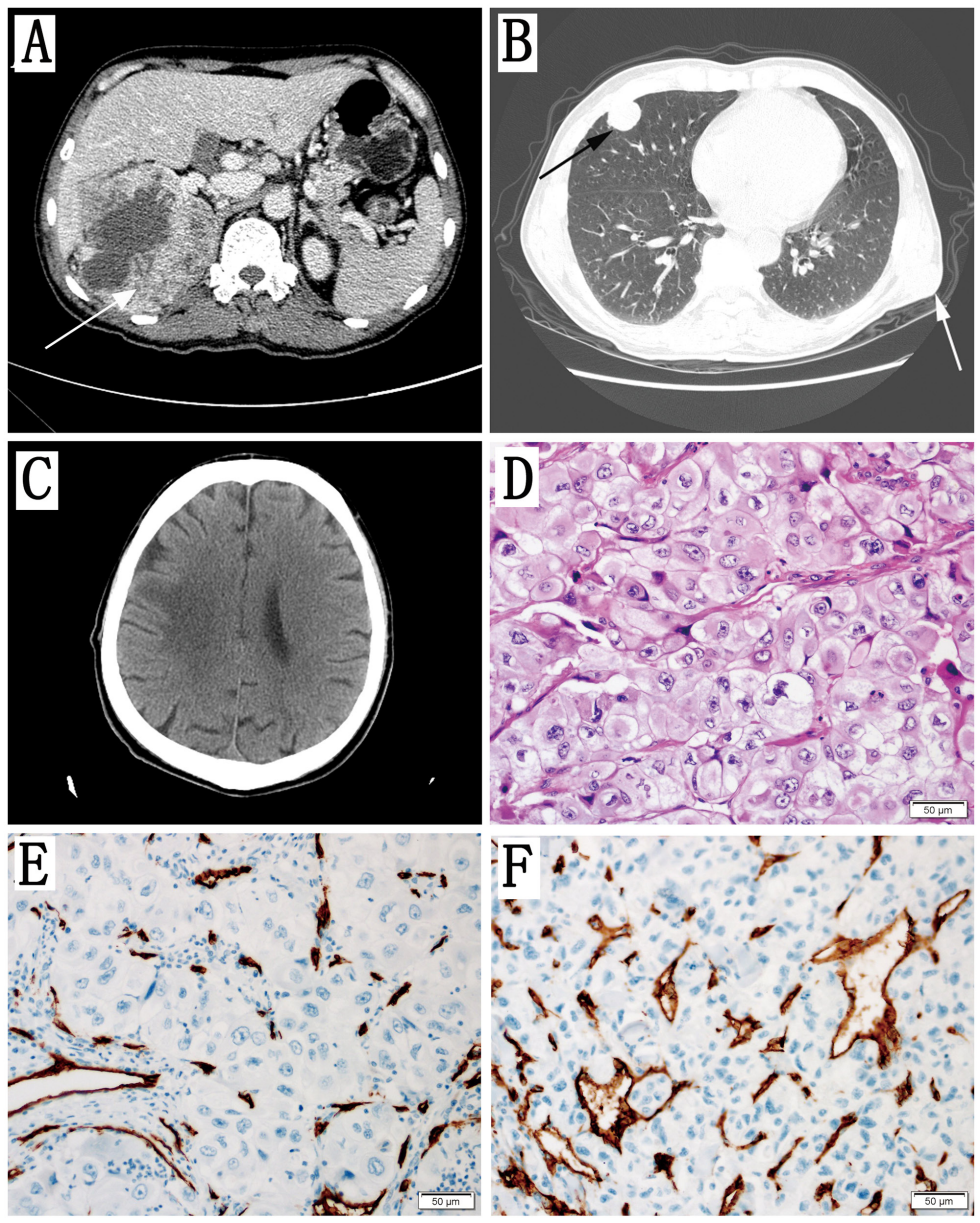
Clinical data of the Patient **(A)** Axial contrast-enhanced CT scan showing a large mass in the right kidney (arrow). **(B)** Axial noncontrast CT scan showing multiple metastases in the lung (black arrow) and one metastasis in the chest wall (white arrow). **(C)** Axial noncontrast CT scan showing brain metastases. **(D)** The pathology results reported that both primary the tumour of the kidney and metastatic tumour of the chest wall were ccRCC. The Fuhrman grade was 4. **(E)** Vascular density stained with CD34 in the primary renal tumour. **(F)** Vascular density stained with CD34 in the subcutaneous metastatic tumour of chest wall.

This 56 years old male patient had a right renal tumour (Figure 3A) with a subcutaneous soft tumour in his left chest wall (Figure 3B) and multiple pulmonary and brain metastases (Figure 3, 3B and 3C). An open radical nephrectomy was performed using the subcostal flank approach, and the subcutaneous metastatic tumour in left chest wall was resected as well. ccRCC metastases were found in the 2 lymph nodes of the 6 resected kidney pedicle lymph nodes. The peritoneum was also invaded by the tumour. The adrenal was normal. The pathological type was ccRCC (Figure 3D). The Fuhrman grade was 4 and the pathological stage was pT4N1M1. Immunohistochemical staining of vascular density (CD34) is shown in Figure 3 (3E and 3F).

### Treatment of PDX lines with targeted therapies

Treatment for mice was initiated when the tumours from 2 ccRCC PDX models reached 100 mm^3^ to 150 mm^3^. The established PDX models were KI2367 from the primary tumour and KI2368 from the subcutaneous metastatic tumour of the chest wall. Each PDX model was divided into 6 experimental groups with 4 mice in every group. The control group (Group 1) was treated orally with vehicle daily (QD)*4 weeks, and the treatment groups were treated with one of following dosing regimens: Sorafenib (Group 2, 50 mg/kg p.o. QD*4 weeks, Dalian Meilun Biotech Co., Ltd), Sunitinib (Group 3, 60 mg/kg p.o. QD*4 weeks, Dalian Meilun Biotech Co., Ltd), Axitinib (Group 4, 15 mg/kg, p.o. QD*4 weeks, Dalian Meilun Biotech Co., Ltd), Sorafenib 50 mg/kg+sunitinib 60 mg/kg p.o. (Group 5, QD*4 weeks) and Sorafenib 50 mg/kg and Sunitinib 60 mg/kg in alternation (Group 6, Weeks 1 and 3, dose with Sunitinib QD, p.o.; Weeks 2 and 4, dose with Sorafenib QD, p.o.). The %ΔT/ΔC value was calculated for assessing the tumour response to the treatment. (%ΔT/ΔC= (mean(T)-mean(T0))/(mean(C)-mean(C0)) *100%, T- Treatment group value, T0 - Treatment group initial value, C - control group value, and C0 - control group initial value). The tumour growth inhibition (TGI) = (1-%ΔT/ΔC))*100.

The mice were kept in individually ventilated cage (IVC) systems at constant temperature and humidity, with 4 animals in each cage. The data for tumour growth and normal behaviour including mobility, visual estimation of food and water consumption, body weight gain/loss measured twice weekly, eye/hair matting and any other abnormal effects, were collected every 3 to 4 days. Tumour sizes were measured every 3 to 4 days using the formula: V = 0.5 length x width^2^. Samples of mice treated with vehicle were stored for future analysis. Any remaining tumour was re-implanted for maintenance of a PDX model.

### Genomic analysis of PDXs

Snap frozen PDX tumour samples were used to extract RNA for transcriptome sequencing (RNA-seq). The purity and integrity were checked with an Agilent Bioanalyzer prior to RNA sequencing. Only RNA samples with RIN >7, 28S/18S >1 and mouse content <10% were used for library construction and sequencing. The sequencing was performed at PE125 on an Illumina HiSeq2500 platform (KI2367) and PE100 on an Illumina HiSeq2500 platform (KI2368) by certified Illumina service providers. The RNAseq raw data were first cleaned by removing reads that were preferentially mapped to a mouse genome (UCSC MM9). Transcript expression was estimated by MMSEQ [63] and represented by log_2_(FPKM). SNP and INDELs were detected by STAR [64] mapping software and GATK [64] variant discovery toolkit, and gene fusions were detected by SOAP fuse [65] and deFuse [66].

### Statistical analysis and software

All statistical analyses were performed using SPSS (IBM Corp. Released 2011. IBM SPSS Statistics for Windows, Version 20.0. Armonk, NY: IBM Corp.). Two sample t-tests were used to check the equality of means between two groups. One-way ANOVA was used to check the equality of means among 3 and more groups. Differences were considered statistically significant at P< 0.05.

## SUPPLEMENTARY MATERIALS FIGURES AND TABLES






