# Supplementary material for: Intra-tumour molecular heterogeneity of clear cell renal cell carcinoma reveals the diversity of the response to targeted therapies using patient-derived xenograft models
Source: Oncotarget. 2017 May 10;8(30):49839–50. doi: 10.18632/oncotarget.17765 (PMC5564811; doi:10.18632/oncotarget.17765)
Supplement: Supplementary file 1 [file oncotarget-08-49839-s001.pdf]

Intra-tumour molecular heterogeneity of clear cell renal cell carcinoma reveals the diversity of the response to targeted therapies using patient-derived xenograft models

SUPPLEMENTARY MATERIALS

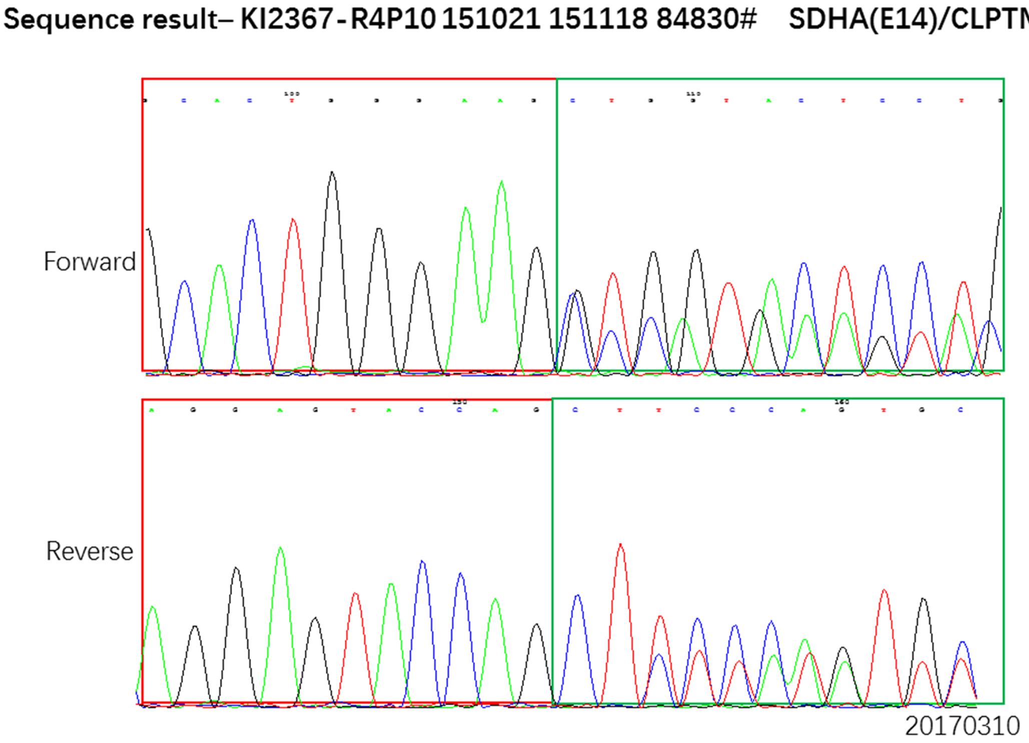

Supplementary Figure 1: Validation for in-frame gene fusions of SDHA/CLPTM1L in KI2367.

Sequence result– KI2368-R4P9 160425 160526 53057# SDHA(E14)/CLPTM1L(E3)

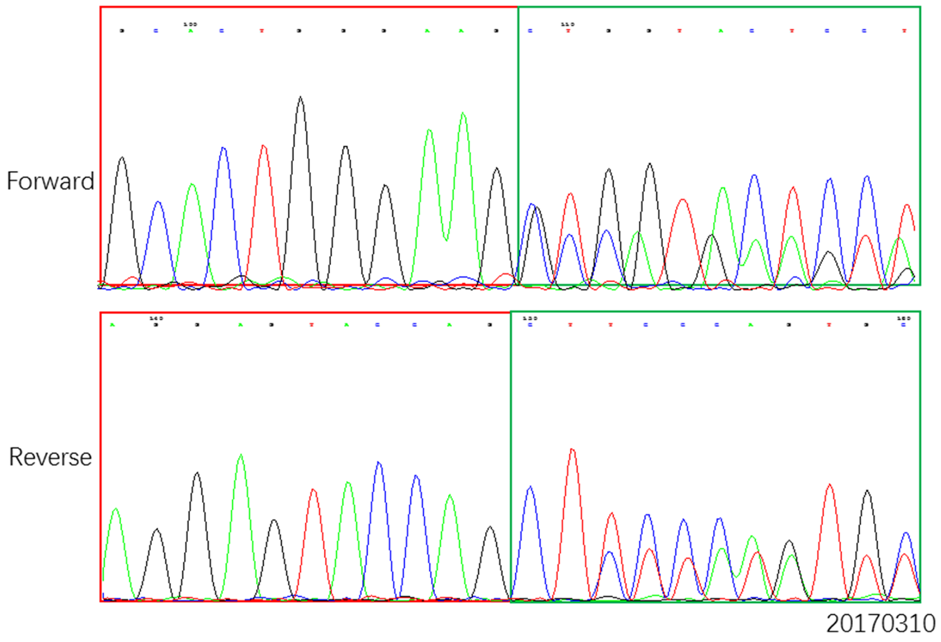

Supplementary Figure 2: Validation for in-frame gene fusions of SDHA/CLPTM1L in KI2368.

Sequence result– KI2368-R4P9 160425 160526 53057# HDAC8/CITED1

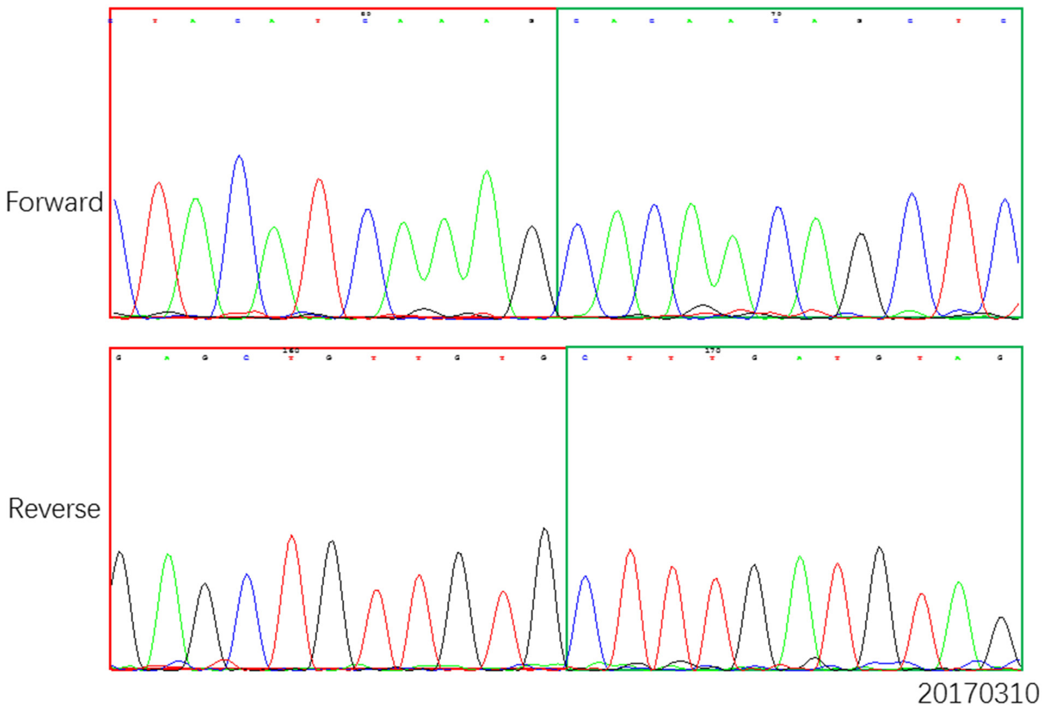

Supplementary Figure 3: Validation for in-frame gene fusions of HDAC8/CITED1.

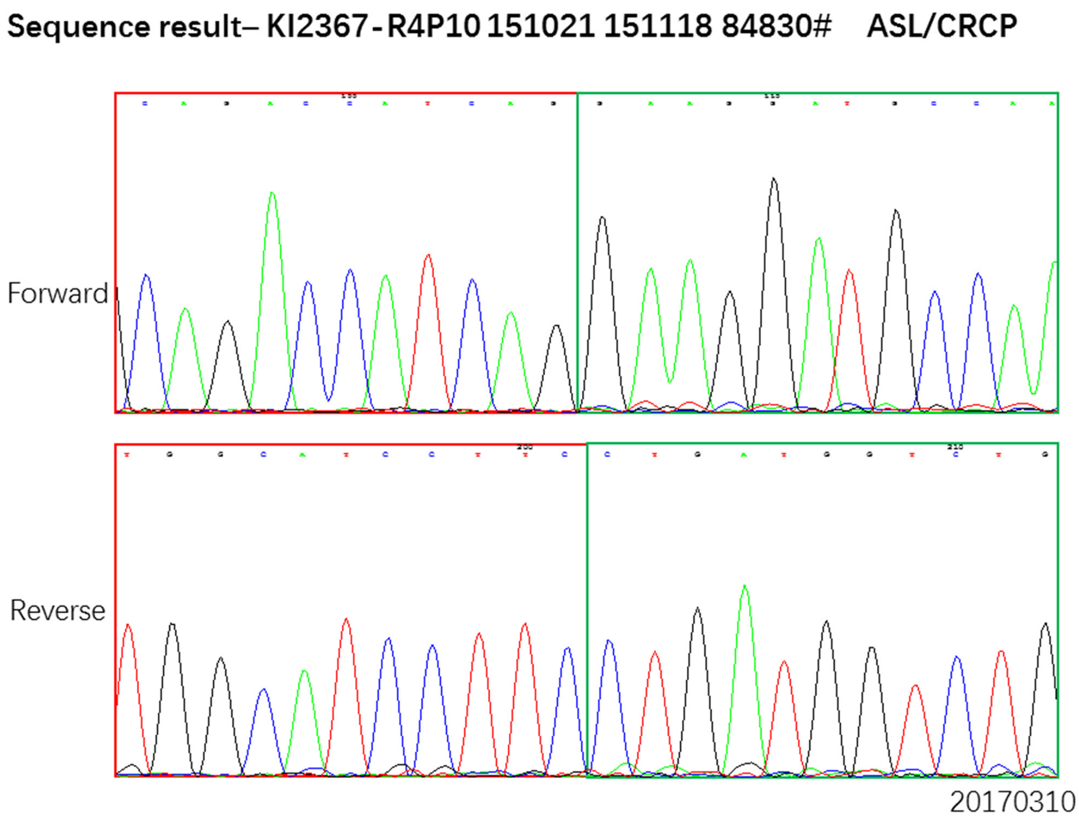

Supplementary Figure 4: Validation for in-frame gene fusions of ASL/CRCP.

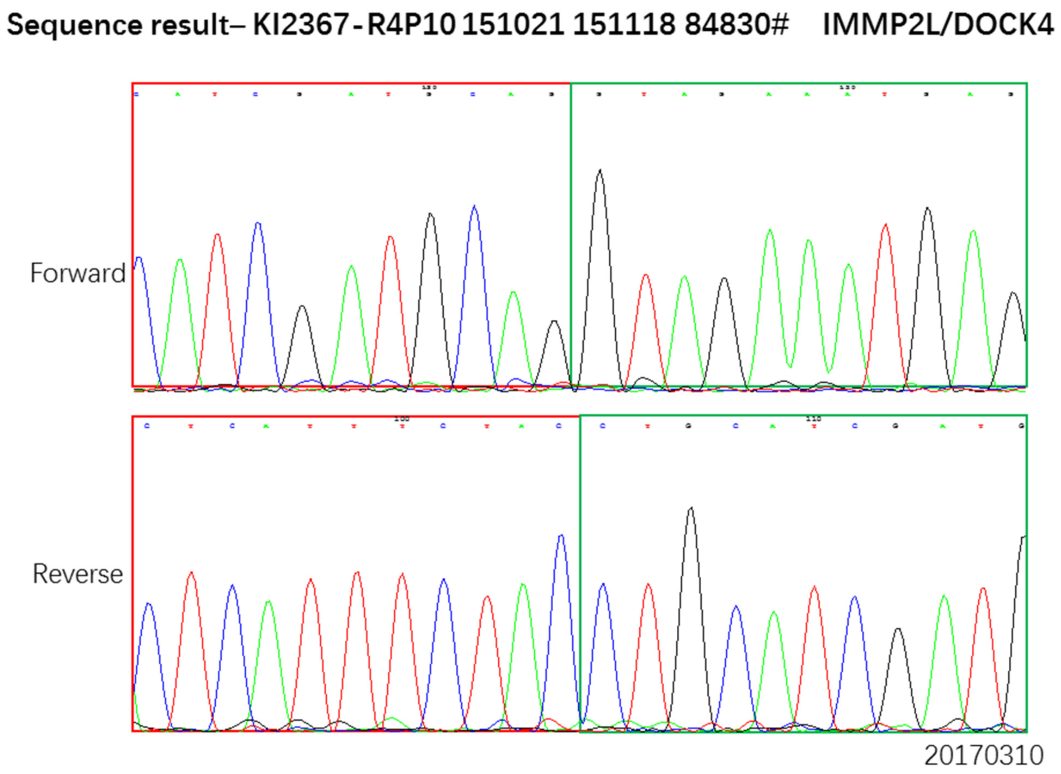

Supplementary Figure 5: Validation for in-frame gene fusions of IMMP2L/DOCK4.

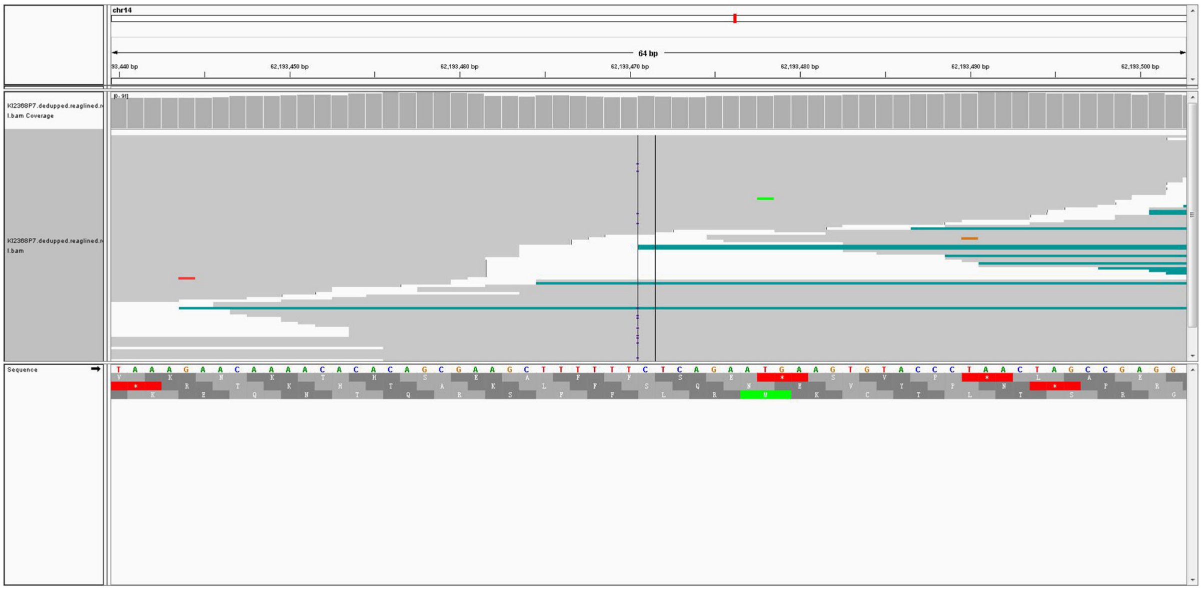

Supplementary Figure 6: HIF1A frameshift mutation in KI2368.

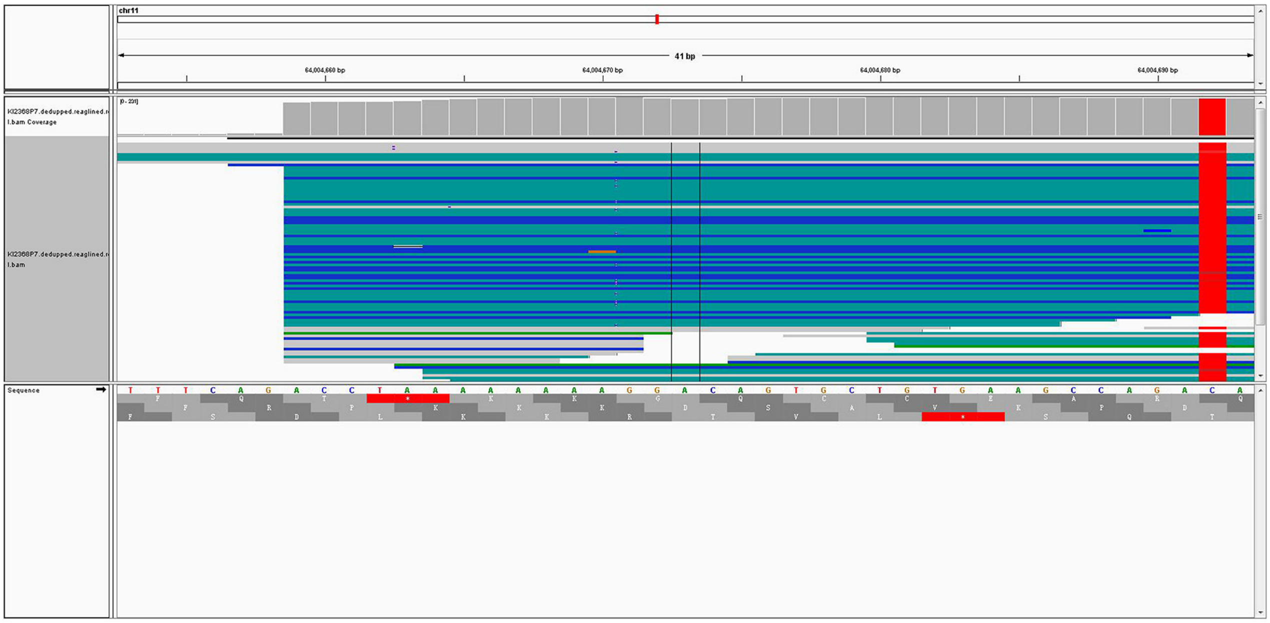

Supplementary Figure 7: VEGFB frameshift mutation in KI2368.

Supplementary Table 1: 1725 genes with > 5-fold higher expression levels in KI2367 than in KI2368.

See Supplementary File 1

Supplementary Table 2: 994 genes had > 5-fold higher expression levels in KI2368 than in KI2367.

See Supplementary File 2
